# An Exploratory Study of the Short‐Term Association of Resistance Training With Circulating Inflammatory Biomarkers, Plasma Brain‐Derived Neurotrophic Factor, and Cognitive Function in Healthy Midlife Adults

**DOI:** 10.1002/brb3.71723

**Published:** 2026-08-25

**Authors:** Yu Ziyan, Chen Deqin, Chen Fanyue

**Affiliations:** ^1^ School of Humanities Shunde Vocational and Technical University Foshan Guangdong China; ^2^ College of Physical Education Beibu Gulf University Qinzhou Guangxi China

**Keywords:** executive function, inflammation, plasma brain‐derived neurotrophic factor, resistance training

## Abstract

**Background:**

Systemic low‐grade inflammation and subtle executive‐function decline can emerge during midlife, yet short‐term lifestyle approaches remain understudied. This single‐arm interventional pre–post study examined whether a 4‐week supervised resistance‐training program was associated with changes in circulating inflammatory biomarkers, plasma brain‐derived neurotrophic factor (BDNF), and executive‐task performance in healthy midlife adults.

**Methods:**

Five hundred healthy adults aged 50–65 years entered a supervised resistance‐training program conducted three times weekly for 4 weeks after screening 647 individuals. Assessments were completed at baseline and 72 h after the final training session. Primary full‐cohort outcomes included plasma interleukin‐6 (IL‐6), tumor necrosis factor‐alpha (TNF‐α), BDNF, two‐back accuracy, two‐back reaction time, Stroop interference, and Stroop accuracy. Adherence was recorded prospectively and summarized as ≥11 sessions (*n* = 312), 10 sessions (*n* = 146), and <10 sessions (*n* = 42). Full‐cohort analyses were treated as the primary analyses, while per‐protocol, adherence‐stratified, and sensitivity analyses were reported as secondary analyses.

**Results:**

In the full analytic cohort, IL‐6 decreased from 2.83 ± 1.60 to 2.09 ± 1.34 pg/mL, TNF‐α decreased from 3.61 ± 1.07 to 3.12 ± 0.96 pg/mL, and BDNF increased from 23.4 ± 5.6 to 26.1 ± 6.2 ng/mL. The corresponding mean changes were −0.74 pg/mL for IL‐6, −0.49 pg/mL for TNF‐α, and +2.7 ng/mL for BDNF, with all paired comparisons statistically significant. Executive‐task performance also improved, with two‐back accuracy increasing from 83.7 ± 8.9% to 87.6 ± 7.2%, two‐back reaction time decreasing from 690 ± 89 to 652 ± 82 ms, Stroop accuracy increasing from 89.8 ± 7.4% to 91.9 ± 6.2%, and Stroop interference decreasing from 238 ± 47 to 211 ± 41 ms. Secondary adherence‐stratified analyses showed larger numerical biomarker and cognitive changes among participants completing ≥11 sessions, but these subgroup findings were interpreted descriptively because adherence groups were not randomized.

**Conclusion:**

In healthy midlife adults, a 4‐week supervised resistance‐training program was associated with lower circulating IL‐6 and TNF‐α, higher plasma BDNF, and modest improvement in working‐memory and inhibitory‐control performance. These findings reflect within‐cohort pre–post associations rather than comparative efficacy and should be interpreted cautiously because of the single‐arm design, absence of central biomarkers, and short follow‐up duration.

## Graphical abstract

Healthy adults aged 50‐65 completed a 4‐week supervised RT programme with plasma biomarkers and executive tasks assessed 72 h after the final session. IL‐6 and TNF‐? decreased, BDNF increased, and task performance improved. Larger shifts appeared with higher adherence. Findings indicate within‐cohort associations requiring controlled mechanistic follow‐up and durability testing.

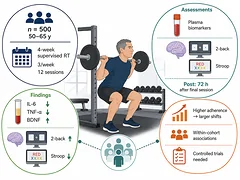

## 1 Introduction

Short‐term resistance training (RT) refers to structured strength‐focused exercise protocols of fewer than 6 weeks that are designed to induce early physiological adaptation before long‐term structural hypertrophy is expected (Fonseca et al. 2025). Its relevance extends beyond strength and mobility because RT may also influence systemic inflammatory pathways, neurotrophic signaling, and cognitive performance during midlife (Babu et al. 2022). Short‐duration protocols are especially pertinent in aging and clinical research because feasibility, supervision, compliance, and safety remain central considerations when previously inactive adults are enrolled in structured exercise programs (Rahmati et al. 2022). Early immune and neuromolecular changes may also precede more durable structural or behavioral adaptations, making brief RT protocols useful for examining short‐interval biological responsiveness (Kurmyshev et al. 2022). In healthy adults aged 50–65 years, this perspective shifts emphasis from long‐term strength remodeling to whether a standardized short‐cycle RT exposure is accompanied by measurable changes in peripheral biomarkers and executive‐task performance.

The biological relevance of RT can be organized through inflammatory and neurotrophic pathways. Interleukin‐6 (IL‐6) and tumor necrosis factor‐alpha (TNF‐α) are commonly used peripheral indicators of systemic inflammatory tone, while brain‐derived neurotrophic factor (BDNF) is a circulating neurotrophic marker associated with plasticity‐supportive processes and cognitive resilience (Wang et al. 2022; Bekinschtein et al. 2014). BDNF has also been linked to executive‐function maintenance and related neurocognitive processes in human studies examining peripheral neurotrophic signaling (Azevedo et al. 2023). However, mechanistic interpretation remains limited because much of the pathway‐level evidence comes from animal models, disease‐specific populations, or multimodal interventions rather than healthy midlife adults exposed to RT alone (Wang et al. 2023; Nakanishi et al. 2023). Clinical multimodal programs have reported reduced systemic inflammatory burden, but they do not isolate the independent contribution of RT or clarify how peripheral biomarker shifts align with cognitive change under a short‐duration resistance‐only protocol (Ayari et al. 2023).

Interpretation of peripheral biomarkers also requires methodological caution. Plasma BDNF concentrations are sensitive to specimen type, platelet storage and release, and pre‐analytical handling; therefore, plasma BDNF is best treated as an indirect circulating neurotrophic biomarker rather than a direct index of central neurobiology (Serra‐Millàs 2016). Neuroinflammation, by contrast, refers to immune signaling within the brain and spinal cord and is typically evaluated using cerebrospinal‐fluid biomarkers, brain tissue indices, neuroimaging proxies, or blood–brain barrier assessments (DiSabato et al. 2016). Because such central indices were not collected in the present study, plasma IL‐6 and TNF‐α are interpreted as systemic inflammatory biomarkers, and plasma BDNF is interpreted as a peripheral neurotrophic biomarker. This distinction allows the biological outcomes to be framed as circulating correlates with plausible neurocognitive relevance rather than as direct measures of central inflammatory or plasticity‐related mechanisms.

Prior evidence supports a conceptual link between inflammatory activity and cognition, but the relationship has not been resolved within short‐cycle RT designs in healthy midlife adults. Inflammation‐related hippocampal disruption has been associated with deficits in executive function and working memory, while elevated midlife IL‐6 and TNF‐α have been linked to later cognitive decline (Li et al. 2025; Kipinoinen et al. 2022). Systemic inflammation has also been associated with poorer verbal fluency, attentional performance, and lower Mini‐Mental State Examination scores, and pro‐inflammatory indices such as the neutrophil‐to‐lymphocyte ratio have been linked to progression from mild cognitive impairment to dementia (Gertje et al. 2023; Giudici et al. 2023; Tondo et al. 2023). These studies establish the biological relevance of the inflammatory–cognitive axis, but they do not determine whether short‐term RT in cognitively healthy midlife adults produces synchronized change in IL‐6, TNF‐α, BDNF, and executive‐task performance within one analytic framework.

Short‐term RT evidence remains heterogeneous because inflammatory, neurotrophic, and cognitive endpoints are often assessed under different sampling schedules and intervention structures. Acute high‐volume RT can produce transient post‐exercise elevations in IL‐6 and TNF‐α, whereas repeated resting assessments may show lower baseline inflammatory concentrations after training exposure (Beisheim‐Ryan et al. 2025; Enes et al. 2025). RT trials centered on functional outcomes often document performance gains without parallel inflammatory measurement, leaving the biological context of improvement incompletely characterized (Fonseca et al. 2025). Combined or multimodal training studies have reported biomarker changes, but the inclusion of aerobic, cognitive, or other exercise components prevents clear attribution to RT alone (Damasio et al. 2025; Wang et al. 2025). These differences show that timing of blood collection, modality composition, and endpoint selection are central sources of inconsistency in the RT biomarker literature.

BDNF‐related RT evidence is similarly uneven. Foundational neurobiological work establishes BDNF as relevant to learning and plasticity‐related signaling, but it does not test short‐term RT exposure in healthy midlife adults (Bekinschtein et al. 2014). RT‐based exercise studies have reported increases in circulating BDNF alongside improved executive outcomes, yet such findings often remain difficult to compare because protocols differ in intervention duration, exercise intensity, cognitive testing structure, and sample characteristics (Lee et al. 2025). Methodological evidence further indicates that specimen handling can substantially affect peripheral BDNF values, reinforcing the need for standardized collection, processing, and assay procedures in RT studies targeting cognition (Serra‐Millàs 2016). A synchronized design that measures plasma BDNF alongside IL‐6, TNF‐α, and executive‐task outcomes can therefore improve interpretive consistency while avoiding direct claims about central BDNF activity.

The cognitive literature linked to RT also remains subdomain‐specific and methodologically fragmented. RT‐focused studies suggest that inhibition, updating, and set‐shifting do not respond uniformly across executive domains, indicating that broad claims of generalized executive enhancement require caution (Jerez‐Salas et al. 2025; Mateluna‐Núñez et al. 2025). Dual‐task, technology‐supported, multimodal, and clinical intervention designs have reported concurrent physiological and cognitive responses, but these protocols often include cognitive loading, aerobic activity, rehabilitation components, or clinical populations that obscure the independent role of RT (Araújo et al. 2025; Gonzalez‐Moreno et al. 2025). Observational evidence linking IL‐6 and TNF‐α to cognitive performance further supports the relevance of inflammatory biomarkers, but it cannot establish within‐participant cognitive change after RT exposure (Gao 2025; Schmitz et al. 2025). Imaging‐oriented and supplementation‐based studies suggest possible fronto‐executive or inflammatory moderation pathways, yet their designs do not align directly with short‐duration RT‐only exposure and synchronized peripheral biomarker measurement (Bonilla et al. 2024; Gu et al. 2024). Recent methodological work has therefore emphasized the need for synchronized biomarker and executive testing within standardized short‐duration exercise frameworks (Mazreno and Taghian 2024).

A consolidated gap emerges across these strands of evidence. Prior studies have not consistently combined a standardized short‐duration RT‐only protocol, delayed post‐intervention biomarker sampling, plasma IL‐6, TNF‐α, and BDNF assessment, and task‐specific executive‐function testing within one healthy midlife cohort. Existing designs also provide limited evidence on whether biomarker change and cognitive‐task change co‐occur across adherence strata when all participants complete baseline and post‐intervention outcome assessments but differ in session completion. This gap is especially relevant because healthy midlife adults may begin with normal‐range inflammatory values and relatively high cognitive performance, making short‐term biological and behavioral changes more difficult to interpret than in clinical or high‐risk samples.

**FIGURE 1:**
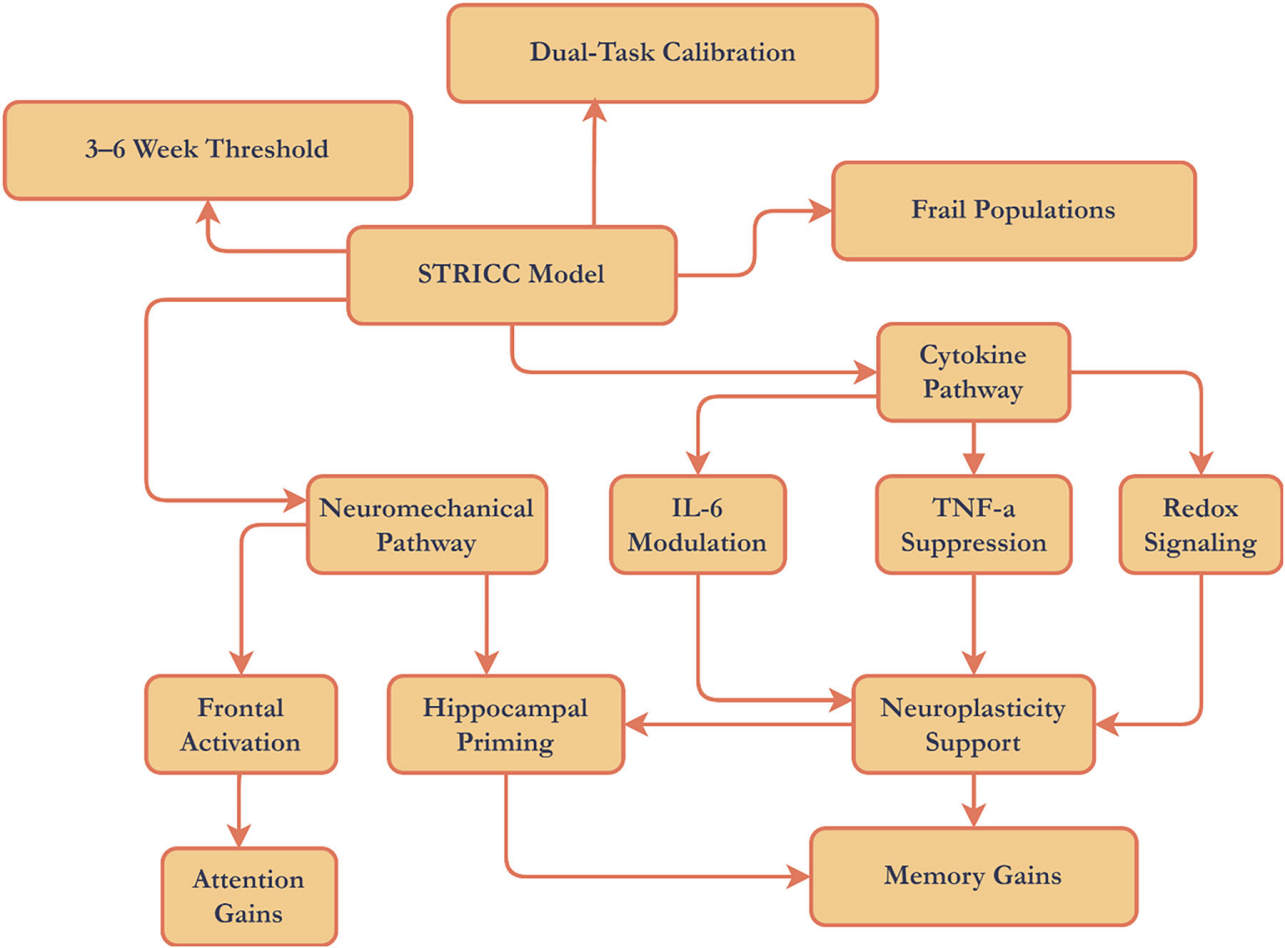
Conceptual structure of the short‐term resistance–inflammation–cognition coupling (STRICC) model, showing a proposed 3–6‐week RT threshold linked to two coordinated pathways: a neuromechanical pathway involving frontal activation, hippocampal priming, attention gains, and memory gains, and a cytokine pathway involving IL‐6 modulation, TNF‐α suppression, and redox signaling converging on neuroplasticity support; dual‐task calibration and frail populations are included as contextual modifiers of pathway expression.

The present study applies the short‐term resistance–inflammation–cognition coupling (STRICC) model as a conceptual framework for organizing synchronized measurement of peripheral inflammatory markers, plasma BDNF, and executive‐task performance (Figure 1). STRICC comprises three linked domains: a peripheral inflammatory component indexed by IL‐6 and TNF‐α, a neurotrophic component indexed by BDNF, and a behavioral executive‐performance component indexed by working‐memory and inhibitory‐control tasks. The model is used to guide endpoint selection and interpretation of co‐occurring changes after a standardized 4‐week RT exposure. It is not used as a mediation model, causal pathway, temporal sequencing model, or direct central nervous system mechanism. Within this framework, dual‐task calibration refers to the conceptual alignment of resistance load and cognitive demand; however, the present intervention used a single‐modality RT protocol, with cognitive outcomes assessed separately at baseline and post‐intervention.

### 1.1 Objectives

This study aims to evaluate whether a 4‐week supervised RT protocol is associated with pre‐ to post‐intervention changes in plasma IL‐6, TNF‐α, and BDNF, and whether these biomarker shifts co‐occur with changes in working‐memory and inhibitory‐control performance among healthy midlife adults. Secondary analyses examine whether these changes differ descriptively across prospectively recorded adherence strata, while maintaining interpretation within the limits of a single‐arm pre–post design.

## 2 Methodology

### 2.1 Study Design

This study was conducted as a single‐center, single‐arm interventional pre–post study at the Human Performance Laboratory, College of Physical Education, Beibu Gulf University, Qinzhou, Guangxi, China. All biomarker and cognitive outcomes were collected at two fixed timepoints: baseline (Week 0) and post‐intervention follow‐up conducted 72 h after completion of the final Week 4 training session. The study was designed to characterize within‐participant short‐term change over a standardized 4‐week exposure period and to examine whether change in plasma biomarkers co‐occurred with change in executive‐task performance under controlled laboratory conditions; it was not designed to estimate comparative efficacy against a concurrent control condition or to support causal inference regarding long‐term preventive effects. Laboratory conditions were standardized across participants for temperature (22–24°C), relative humidity (40%–55%), and light intensity (350–400 lux). All baseline blood sampling and cognitive testing were completed between 7 and 9 a.m. after at least 10 h of fasting and at least 12 h of abstinence from exercise, caffeine, and alcohol; the same time window and pre‐assessment restrictions were used at post‐intervention follow‐up. All acquisition procedures followed a locked standard operating protocol (SOP v1.2) finalized before recruitment and archived.

The intervention duration was fixed at 4 weeks and consisted of 12 supervised resistance‐training sessions delivered on three non‐consecutive days per week. RT was implemented as a standardized, moderate‐load, whole‐body protocol using compound movements selected to provide consistent neuromuscular exposure across large muscle groups while minimizing technical failure risk in previously inactive adults aged 50–65 years. Detailed loading parameters, rating of perceived exertion procedures, rest intervals, exercise order, and progression rules are reported in Section 2.3.3 and Section 2.3.5. The study was therefore structured to examine synchronized pre–post change in peripheral biomarkers and executive‐task performance after a short‐cycle RT exposure, without repeating exercise‐prescription parameters in the design overview.

STRICC constructs were operationalized using pre‐specified biological and behavioral endpoints collected at baseline (Week 0) and post‐exposure (72 h after the final Week 4 session). The inflammatory component was indexed by plasma IL‐6 and TNF‐α, quantified from fasting venous blood using standardized enzyme‐linked immunosorbent assay procedures with plate‐level quality‐control thresholds. The neurotrophic component was indexed by plasma BDNF quantified under the same pre‐analytic and assay conditions. Executive and working‐memory endpoints were operationalized as 2‐back accuracy, 2‐back reaction time on correct target trials, Stroop interference (incongruent minus congruent reaction time), and Stroop accuracy, computed using pre‐specified trial‐validity thresholds and block‐retest rules. STRICC was used to organize endpoint selection and interpretation of synchronized biomarker and cognitive measurements in the present study; it was not tested as a mediation model, path model, or temporal sequencing model. No cerebrospinal fluid, neuroimaging, blood–brain barrier, or other central nervous system–aligned biomarkers were collected. Within STRICC, dual‐task calibration was treated as a conceptual alignment of cognitive load and physiological state at assessment, whereas cognitive testing in the present study was conducted as a standalone battery under standardized morning conditions and after a fixed washout interval of 72 h post‐final session to minimize acute fatigue and short‐lived post‐exercise cytokine excursions.

#### 2.1.1 Ethical Approval

The study protocol and informed consent procedures received ethical approval from the Institutional Review Board (Approval Code: BGU‐HPL‐2025‐003). Written informed consent was obtained from all participants prior to data collection. The study adhered fully to the principles of the Declaration of Helsinki (2013 revision) and the reporting expectations for non‐randomized pre–post intervention research.

### 2.2 Participants

#### 2.2.1 Recruitment

**FIGURE 2:**
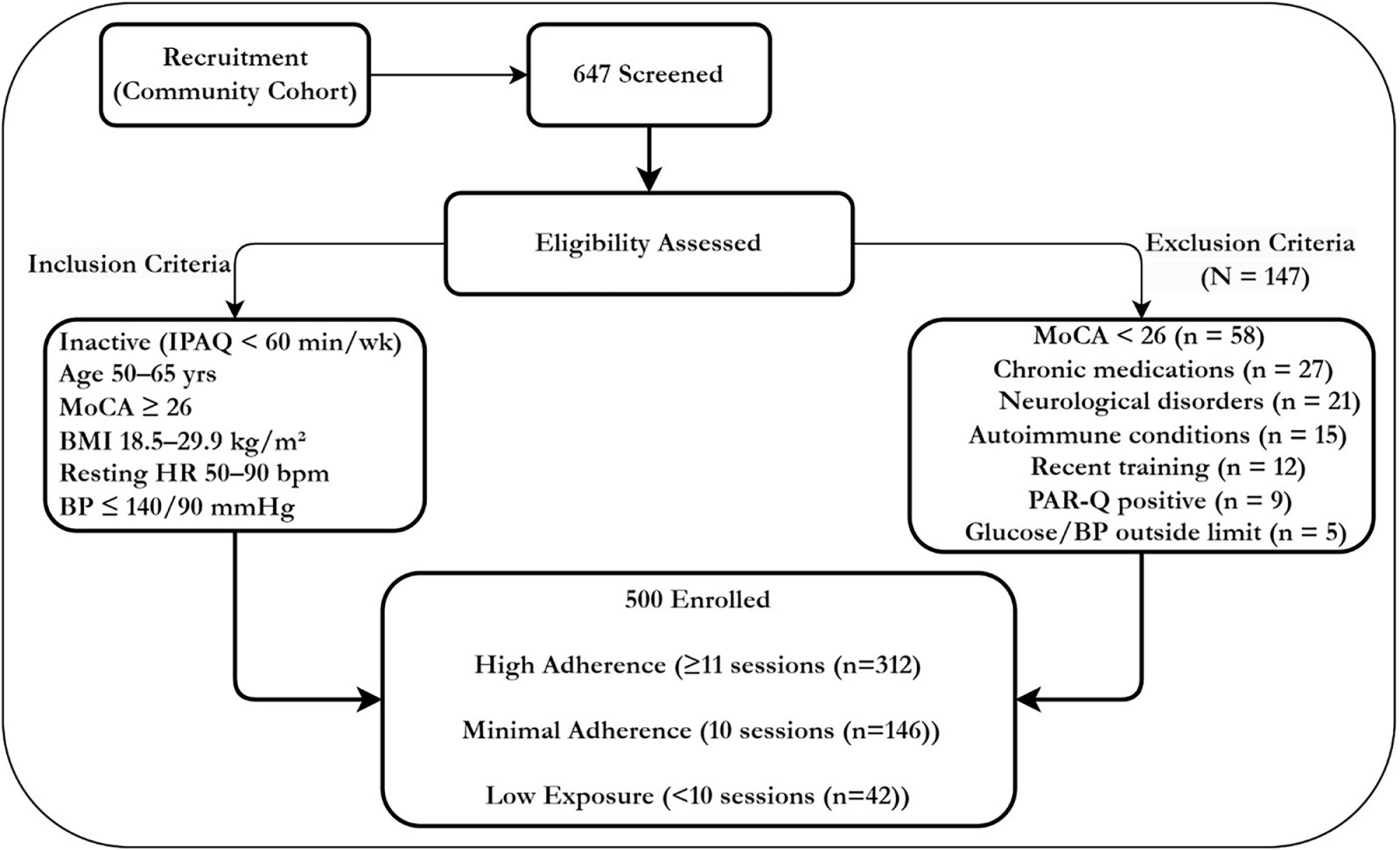
Flow diagram of participant recruitment, eligibility screening, exclusion, enrollment, and adherence classification. A total of 647 individuals from the community cohort were screened; 147 were excluded on cognitive, clinical, training, or physiological eligibility grounds; and 500 were enrolled in the study. The enrolled sample was subsequently classified by session completion as high adherence (≥11 sessions, *n* = 312), minimal adherence (10 sessions, *n* = 146), and low exposure (<10 sessions, *n* = 42).

Participants were recruited between February and April 2025 from community health centers, university notice boards, and regional wellness screening programs in Qinzhou, Guangxi (see Figure 2). All interested individuals completed a structured phone pre‐screen followed by a face‐to‐face eligibility assessment at the Human Performance Laboratory, Beibu Gulf University. A total of 647 individuals were screened; 500 met all eligibility criteria and were enrolled for full protocol adherence. Screening exclusions occurred before enrollment and did not constitute post‐baseline attrition. After enrollment, all 500 participants completed baseline and post‐intervention biomarker and cognitive assessments; adherence variation was therefore expressed as differential session completion within the intervention period rather than loss to outcome follow‐up. Recruitment and implementation followed a rolling cohort schedule between February and April 2025. Candidates were screened in weekly batches of approximately 45–60 individuals from community health‐center referrals, university notices, and regional wellness‐screening programs. Eligible participants were assigned to 20 supervised training cohorts of approximately 20–30 participants, with sessions distributed across morning and late‐afternoon blocks from Monday to Saturday. Baseline biomarker and cognitive assessments were completed 3–7 days before the first session, and post‐intervention assessments were scheduled 72 h after the final Week 4 session. Retention procedures included 24 h telephone or text reminders, flexible make‐up scheduling within the same training week, transport reimbursement for assessment visits, and immediate rescheduling after non‐emergency absence. These procedures supported complete baseline and post‐intervention assessment, while differences in completed sessions were analyzed as adherence variation rather than outcome attrition.

#### 2.2.2 Eligibility Criteria

Years of formal education were recorded at enrollment and prespecified for use as a covariate in adjusted cognitive analyses because educational attainment may influence baseline executive‐task performance. The eligibility framework was intentionally restrictive in order to define a healthy midlife cohort with reduced inflammatory and clinical confounding; the analytic target population was therefore previously inactive adults aged 50–65 years who met the stated safety and cognitive screening criteria, not clinical populations or adults with obesity‐related or multimorbid profiles.

Inclusion criteria were: (i) age between 50 and 65 years (validated via government‐issued ID) (Chodzko‐Zajko et al. 2009); (ii) Montreal Cognitive Assessment (MoCA) ≥ 26 at baseline screening, administered by certified raters using version 7.2 (Nasreddine et al. 2005); only participants meeting this threshold were enrolled; (iii) physically inactive, defined as fewer than 60 min/week of structured exercise across the preceding 12 weeks, verified using a brief Exercise History Inventory (week‐by‐week calendar recall) corroborated by step‐count logs from participants’ devices when available. IPAQ‐SF was used to confirm low activity in the prior 7 days (CRAIG et al. 2003); the Exercise History Inventory established inactivity over 3 months; (iv) body mass index between 18.5 and 29.9 kg/m^2^; this range was selected to reduce baseline heterogeneity in systemic inflammatory status, exercise tolerance, and cardiometabolic risk during a first short‐duration mechanistic RT exposure in previously inactive adults; (v) resting systolic/diastolic blood pressure < 140/90 mmHg recorded on two non‐consecutive days using a calibrated Omron HEM‐7120 (Japan); (vi) resting heart rate 50–90 bpm measured after 10 min seated rest; three successive readings were acquired at 60‐s intervals using a Polar H10 chest strap paired to a validated receiver, with the average of the last two readings recorded.

For cognitive analyses, only enrolled participants with baseline MoCA ≥ 26 contributed data; any individuals scoring < 26 were screen failures and are not included in the analytic cohort. Exclusion criteria were: (i) diagnosed neurological disorders such as stroke, epilepsy, or Parkinson's disease; (ii) clinical history of inflammatory or autoimmune disorders including rheumatoid arthritis and lupus; (iii) current or recent (within 3 months) use of corticosteroids, antidepressants, or other psychotropic medications; (iv) structured RT within the past 12 weeks (to align with the inactivity definition above); (v) medical contraindications as per PAR‐Q+ screening (Bredin et al. 2013) and (vi) fasting blood glucose ≥ 6.9 mmol/L or systolic blood pressure ≥ 160 mmHg during baseline screening.

#### 2.2.3 Power Analysis

A priori power analysis was conducted using G*Power 3.1.9.7. The calculation was anchored to the expected pre–post change in plasma interleukin‐6 (IL‐6), because IL‐6 was the most conservative biomarker endpoint with prior short‐term exercise data available for planning. Assuming a mean IL‐6 reduction of 0.70 pg/mL, standard deviation of 2.50 pg/mL, two‐tailed paired‐sample test, α = 0.05, and power = 0.90, the required sample size was 438 participants. A 12% allowance was added to protect the final analytic sample against expected non‐completion of follow‐up assessment, assay failure, or unusable cognitive‐task blocks, yielding a target recruitment size of 500 participants. Cognitive endpoints, including two‐back accuracy, two‐back reaction time, Stroop interference, and Stroop accuracy, were not used as the basis for the sample‐size calculation because comparable short‐term RT‐only effect estimates in healthy midlife adults were limited. These cognitive outcomes were therefore analyzed as synchronized behavioral endpoints within the same pre–post framework, with interpretation based on effect magnitude, confidence intervals, and false discovery rate (FDR)‐adjusted significance rather than on a separate endpoint‐specific power calculation.

#### 2.2.4 Stratification and Bias

Participants were stratified a priori by age (50–57, 58–65) and by biological sex to balance subgroup counts around the sample median age and to support planned subgroup summaries. Stratification bands were selected to produce two contiguous, non‐overlapping ranges spanning the full 50–65 window. Targeting ages 50–65 captures the midlife–early older adult transition when brain and neuromuscular trajectories begin to shift, BDNF levels tend to decline, and inflammation–cognition links emerge, while remaining below typical geriatric frailty thresholds (Fried phenotype). This window is also widely used in resistance‐training trials and aligns with major guidance that treats up to ∼65 as “adult” for strength programming, supporting safe, supervised RT with cognitive endpoints (Fragala et al. 2019; Fried et al. 2001; Raz et al. 2010). Selection bias was minimized through pre‐screening by independent assessors uninvolved in exposure implementation. Biomarker technicians and cognitive assessors worked in separate rooms and were masked to timepoint and adherence status using coded alphanumeric IDs. Sample labels contained no dates or session counts. Performance bias was addressed by maintaining uniform exposure conditions across participants, with no variable load manipulation. Cognitive tasks were launched by an administrator unaffiliated with training sessions; the administrator could not access pre/post flags within the software console. Exposure supervisors did not conduct outcome testing. Recall bias was limited through the exclusive use of objectively measured variables (IL‐6, task reaction times). Procedural bias was minimized by timestamping all sample collections and assessments within fixed morning hours (7–9 a.m.), and by enforcing a 10‐h fasting period and a 12‐h exercise abstinence window before data collection. All data entry used double‐blind encoding with audit trail logging in Research Electronic Data Capture (REDCap) (Harris et al. 2009).

In Figure 2, a total of 647 individuals were screened; 147 were excluded based on cognitive, health, or training criteria; and 500 met all inclusion standards. All enrolled participants completed pre‐ and post‐testing. Adherence distribution comprised 312 participants with ≥ 11 sessions, 146 with 10 sessions, and 42 with < 10 sessions. No losses occurred due to assay failure or missing cognitive data.

### 2.3 Exposure Classification Framework

#### 2.3.1 Exposure Definition

The exposure of interest was defined as participation in a standardized, moderate‐load, whole‐body RT regimen over 4 consecutive weeks. Exposure was uniformly administered across all participants to ensure consistent classification and mechanistic alignment with the STRICC model's neuromechanical and cytokine‐adaptive pathways.

#### 2.3.2 Exposure Session Structure

The exposure session structure consisted of three non‐consecutive sessions per week, conducted on alternating weekdays (Monday, Wednesday, and Friday). Each session lasted approximately 50 min and included a 5‐min dynamic warm‐up, a 35‐min resistance circuit, and a 10‐min cool‐down phase. All sessions were held at the Human Performance Laboratory's supervised strength training zone under ambient‐controlled conditions. No unsupervised training or home‐based physical activity was permitted during the study period.

#### 2.3.3 Exercise Prescription and Intensity

Each session comprised six compound exercises targeting major muscle groups: leg press, chest press, seated row, lat pulldown, bodyweight squat, and static plank holds. Machine‐based exercises were performed on selectorized resistance equipment with adjustable load increments. Initial exercise load was prescribed at 60%–70% of estimated one‐repetition maximum (1RM), calculated from a submaximal familiarization test conducted during the baseline training‐orientation visit. Participants completed three sets of 10–12 repetitions for each dynamic exercise. Session intensity was monitored using the Borg 6–20 rating of perceived exertion (RPE) scale, with a target RPE range of 12–14 during work sets, corresponding to moderate perceived exertion (Borg 1982, 1998). Static plank holds were performed for three sets of 30 s each. Rest intervals were fixed at 60–90 s between sets and exercises, and movement tempo for bodyweight squats was standardized at 3:1:1 for eccentric, pause, and concentric phases. Static core holds were performed for three sets of 30 s each. Rest intervals were fixed at 60–90 s between sets and exercises. For bodyweight movements, tempo was standardized at 3:1:1 (eccentric–pause–concentric), and all movements were full range of motion under trainer supervision.

#### 2.3.4 Session Structure and Supervision

Each session was supervised by certified strength and conditioning specialists with a minimum of NSCA‐CSCS or equivalent national certification (Garber et al. 2011). A standardized supervision protocol was followed using session checklist logs. Trainers provided verbal cues, corrected technique, and ensured consistent load documentation. Trainer‐to‐participant ratio was maintained at 1:5. Inter‐session variability was controlled by assigning each participant to the same trainer across all sessions, and by using pre‐programmed resistance machine load guides to avoid manual misadjustment.

#### 2.3.5 Progression and Load Management

Progression was evaluated during the first set of each exercise at the first session of each training week. When a participant completed ≥12 repetitions with stable technique and RPE ≤12, the external load for that exercise was increased by 5% at the next session. When repetitions fell below 10, or RPE was ≥15, the load was reduced by 2.5%–5% to maintain the prescribed moderate‐intensity range. When neither progression nor reduction criteria were met, the load was held constant. Weekly load adjustment followed American College of Sports Medicine (ACSM) progression guidance recommending small stepwise increases when the prescribed repetition range is exceeded, operationalized in this protocol as a +5% increment after technically stable completion of the upper repetition target (American College of Sports Medicine 2009). This approach aligns with ACSM progression models and with autoregulatory paradigms of Autoregulatory Progressive Resistance Exercise (APRE) and repetitions‐in‐reserve (RIR) frameworks that individualize loading to observed performance (Helms et al. 2016; Mann et al. 2010). All changes were recorded in the load log. If progression criteria were not met, the load remained unchanged. No deload or taper phases were used, as the short duration and mechanistic focus on neuromuscular adaptation and inflammation did not require periodization. The protocol intentionally avoided hypertrophic emphasis to isolate neuromechanical and cytokine mechanisms.

#### 2.3.6 Adherence and Protocol Fidelity

Adherence was defined a priori as completion of ≥10 of 12 sessions (≥83%). Attendance was recorded prospectively at every scheduled session using trainer‐completed logs cross‐checked against participant sign‐in records and daily scheduling sheets. Session completion was also retained as a continuous count from 0 to 12 for descriptive tracking of exposure completion. Of the 500 enrolled participants, 458 (91.6%) met the prespecified adherence threshold and constituted the per‐protocol subset. The remaining 42 participants completed fewer than 10 sessions and were retained in full‐cohort analyses. For descriptive adherence stratification, participants were also grouped as ≥11 sessions (*n* = 312), 10 sessions (*n* = 146), and <10 sessions (*n* = 42). These adherence strata were treated as non‐randomized exposure‐completion categories and were not interpreted as randomized dose groups, because higher attendance may correlate with participant motivation, baseline health behavior, and other unmeasured characteristics. The full enrolled cohort of 500 participants, all of whom completed baseline and post‐exposure biomarker and cognitive assessments, was retained for full‐cohort pre–post analyses, whereas adherence‐stratified and per‐protocol analyses were reported as secondary analyses. For each missed session, the immediate reason was recorded in the session log as scheduling conflict, transient illness, temporary musculoskeletal discomfort, or other non‐emergency absence category.

Retention support was standardized across participants and included 24‐h appointment reminders, flexible supervised make‐up scheduling within the same training week, transport reimbursement for assessment visits, and direct follow‐up after missed sessions. These procedures were applied to maintain outcome assessment completion rather than to increase training dose. Because participants who used flexible scheduling or completed more sessions may have differed in motivation, availability, health behavior, or expectancy, adherence strata were treated as observational exposure‐completion categories and not as randomized dosage groups.

Protocol fidelity was confirmed through audit checklists, trainer supervision records, load logs, and rest‐interval monitoring. Adverse events were monitored at each session using a structured safety form documenting musculoskeletal pain exacerbation, dizziness, presyncope, chest symptoms, falls, or any event requiring exercise cessation, modification, or medical review. No adverse events occurred, and no deviations in exposure timing or delivery were recorded.

### 2.4 Biomarker Assays

All biological markers were measured in peripheral blood plasma and reported as circulating concentrations. Plasma IL‐6, TNF‐α, and BDNF were selected to characterize systemic inflammatory tone and peripheral neurotrophic signaling within a short‐duration intervention framework. These assays were not intended to measure central nervous system cytokines, neuroinflammation, blood–brain barrier transport, or central neurotrophic activity. The overview of biomarker interpretation is provided here, while the detailed venipuncture timing, ethylenediaminetetraacetic acid tube handling, centrifugation, aliquoting, storage, assay‐kit specifications, duplicate rules, and quality‐control thresholds are reported in Sections 2.4.1–2.4.3.

#### 2.4.1 Sample Collection and Handling

Fasting venous blood was collected at baseline (Week 0) and 72 h post‐final session (Week 4) between 7 and 9 a.m. Participants fasted for 10 h and abstained from exercise, caffeine, and alcohol for 12 h. Blood was drawn from the antecubital vein using 21G butterfly needles into 5‐mL EDTA tubes. Tubes were inverted 10 times, placed on ice, and transported within 5 min. Plasma was separated by centrifugation at 1500 × *g* for 10 min at 4°C, aliquoted in 0.5 mL triplicates, and stored at −80°C.

#### 2.4.2 Assay Protocol and Kit Specifications

IL‐6, TNF‐α, and BDNF were quantified via high‐sensitivity ELISA. Kits used: IL‐6 (R&D Systems, USA, HS600B), TNF‐α (Abcam, UK, ab46087), and BDNF (Boster, USA, EK0307). Assays followed manufacturer protocols, including incubation, washing, and development steps. Absorbance was read at 450 nm with 540 nm correction using a Thermo Fisher Multiskan Sky spectrophotometer.

#### 2.4.3 Calibration, Replicates, and Quality Control

All samples were assayed in duplicate. If OD values differed by >15%, samples were re‐run. Standard curves (*R* ^2^ ≥ 0.995) were generated per plate. Intra‐assay and inter‐assay CVs were maintained ≤10% and ≤12%, respectively, using pooled plasma controls. Technicians were blinded to timepoints. Pipetting was performed using calibrated Eppendorf Xplorer Plus electronic pipettes. All reagents were equilibrated to room temperature before use. Plates with control CVs exceeding thresholds triggered a full re‐run. Any sample with duplicate CV > 15% after repeat was excluded from analysis for that marker only and treated as missing in the multivariate models.

### 2.5 Cognitive Testing

Cognitive assessment was standardized to reduce procedural variability and to limit, but not eliminate, repeated‐testing effects. The 2‐back and Stroop tasks were administered in the same room, on the same device class, within the same morning assessment window at baseline and post‐intervention follow‐up. Each task included a fixed practice block before scored testing, and scored blocks were completed without performance feedback. Trial order was pseudo‐randomized within each timepoint, invalid blocks were immediately repeated under the same settings according to predefined thresholds, and the post‐intervention assessment was scheduled 72 h after the final training session to reduce acute fatigue effects. Because no alternate‐form cognitive battery and no non‐exercising comparator group were used, residual task familiarization could not be fully excluded, and the cognitive battery should be interpreted as focused assessment of working memory and inhibitory control rather than as a comprehensive measure of all executive domains.

#### 2.5.1 2‐Back Working Memory Task

The 2‐back task indexed updating and online maintenance of working memory and was chosen for its sensitivity to short‐term training effects on executive efficiency (Kirchner 1958; Mathôt et al. 2011). Testing occurred in a quiet, dim room (22–24°C). Stimuli were presented centrally in OpenSesame on a 15.6″ liquid‐crystal display (60 Hz; 1920×1080) at 60–70 cm viewing distance. The stimulus set comprised uppercase consonants excluding visually confusable letters; characters were white on mid‐gray, sans‐serif, ∼1.2° visual angle. Each trial began with a fixation cross (500 ms), followed by a letter (500 ms) and a blank interval (1500 ms), yielding a fixed 2000 ms stimulus‐onset asynchrony. Participants completed three blocks of 40 trials (120 total). A trial was a “target” when the current letter matched the letter two trials earlier (n–2); non‐targets were either lures (n–1 or n–3 matches) or novel non‐matches. Blocks were pseudo‐randomized with ∼33% targets, ∼17% lures (balanced n–1/n–3), and ∼50% novel non‐targets, enforcing a minimum lag of three between targets to prevent anticipation. Responses were made on a single USB key (press on targets only), with standardized instructions emphasizing both speed and accuracy. A 20‐trial guided practice with feedback preceded testing; participants proceeded after achieving ≥70% practice accuracy. If practice accuracy remained below 70% on the first attempt, task instructions were repeated once and a second 20‐trial practice block was administered before the scored blocks were initiated.

Primary outcomes were overall accuracy (percent correct across all trials), median reaction time (ms) on correct target trials, and error decomposition into omissions (missed targets) and commissions (false alarms on non‐targets). Accuracy was computed as (hits + correct rejections) ÷ total trials × 100. Anticipatory keypresses <150 ms and late responses >2500 ms were excluded from reaction‐time calculations and from accuracy denominators for this task only. Blocks with >15% invalid trials (anticipations, timeouts, device errors) were immediately repeated after a 2‐min break under the same settings, and the repeat replaced the invalid block for analysis

#### 2.5.2 Stroop Color–Word Interference Test

The Stroop task indexed inhibitory control and selective attention (MacLeod 1991; Stroop 1935). Testing used the same room and display settings as the 2‐back. Stimuli were color words of RED, BLUE, and GREEN rendered in a sans‐serif font at ∼1.2° visual angle. Three conditions were presented: neutral (strings of “XXXX” in colored ink), congruent (word matches ink), and incongruent (word differs from ink). Ink colors (red, blue, and green) were balanced within each condition. Participants completed 3 blocks × 30 trials (90 total), with an equal mix of neutral, congruent, and incongruent trials per block. Each trial displayed the stimulus for 1000 ms followed by a 1500 ms inter‐trial interval. Condition order and item sequences were pseudo‐randomized with the constraint that no more than two identical conditions occurred consecutively. A three‐button USB pad was used, with each button mapped to one ink color only (left = red, middle = blue, right = green). Mapping was explained on‐screen and rehearsed in practice; participants were instructed to report the ink color, ignoring the word. Before testing, they completed a 12‐trial practice with brief feedback and an Ishihara color‐vision check (participants who failed the Ishihara check would have been excluded from the Stroop analysis set; in this cohort, no participants failed (Cheney et al. 2019). If a participant showed persistent response‐mapping errors during practice, the response mapping was re‐explained, and the practice block was repeated once before scored trials commenced.

Primary outcomes were: (i) interference score = mean reaction time (RT) in incongruent trials − mean RT in congruent trials; (ii) total accuracy (% correct across all trials); and (iii) median RT for correct responses (reported by condition). Trials with RT <200 ms or >2000 ms were excluded from RT and accuracy calculations for this Stroop task only. If a block contained >15% invalid responses (anticipations/timeouts/multiple keys), it was immediately retested after a short break and the retest replaced the invalid block.

### 2.6 Statistical Analysis

All analyses were conducted using R version 4.2.1 (R Foundation for Statistical Computing, Vienna, Austria). Descriptive statistics were reported as mean ± SD for continuous variables and frequency (%) for categorical variables. Normality of outcome distributions was assessed using Shapiro–Wilk tests and Q–Q plots. Biomarker and cognitive outcomes were compared between baseline and post‐exposure using paired‐sample t‐tests for normally distributed data or Wilcoxon signed‐rank tests for non‐normal data.

Primary outcomes were pre–post changes in IL‐6, TNF‐α, BDNF, 2‐back accuracy, 2‐back reaction time on correct target trials, Stroop interference, and Stroop accuracy. The principal inferential objective was estimation of within‐participant change across the 4‐week intervention period in the full enrolled cohort; analyses were therefore framed as pre–post association testing within a single‐arm design rather than as causal effect estimation relative to a comparator group. Pre–post comparisons used paired‐sample *t*‐tests for normally distributed data or Wilcoxon signed‐rank tests for non‐normal distributions. For adjusted subgroup summaries, analysis of covariance was applied with post‐intervention value as the dependent variable, baseline value as covariate, and subgroup factor entered together with years of formal education as an additional covariate for cognitive outcomes. Associations between biomarker change scores and cognitive change scores were assessed using Pearson or Spearman coefficients as appropriate. Adherence‐stratified analyses were prespecified as secondary descriptive and inferential analyses across non‐randomized exposure‐completion categories and were not interpreted as formal dose–response tests. STRICC was not tested using mediation analysis, structural path modeling, or temporal sequencing analysis. Two‐sided α was set at 0.05.

The primary powered endpoint for sample‐size planning was the pre–post change in plasma IL‐6. Tumor necrosis factor‐alpha and BDNF were analyzed as additional biomarker endpoints within the same pre–post biomarker family. Cognitive outcomes were treated as synchronized behavioral endpoints designed to characterize co‐occurring change in working memory and inhibitory control after the same RT exposure. Because the sample‐size calculation was not based on expected cognitive‐task effects, cognitive findings were interpreted using effect estimates, 95% confidence intervals, and FDR‐adjusted results, with emphasis on magnitude and consistency rather than endpoint‐specific power. Adherence‐stratified analyses were interpreted as secondary non‐randomized exposure‐completion summaries because attendance may reflect scheduling flexibility, motivation, baseline health behavior, or other unmeasured factors.

To control multiplicity within related endpoint families, FDR adjustment was applied using the Benjamini–Hochberg (BH) procedure with *q* = 0.05. BH correction was performed separately for two primary families: (i) biomarker endpoints (IL‐6, TNF‐α, and BDNF) derived from paired pre–post comparisons; and (ii) cognitive endpoints (2‐back accuracy, 2‐back reaction time, Stroop interference, and Stroop accuracy) derived from paired pre–post comparisons. For each family, unadjusted *p* values were ranked from smallest to largest, BH‐adjusted *q* values were computed, and significance was assigned when *q* ≤ 0.05 for the corresponding endpoint within that family. For correlation analyses between biomarker change scores and cognitive change scores, BH control was applied within the set of correlation tests reported together as a distinct family to maintain interpretability of association screening. Statistical outputs retained both the unadjusted *p* value and the BH‐adjusted *q* value for each endpoint to distinguish nominal from FDR‐controlled significance.

Effect sizes were reported as Cohen's *d* (parametric) or matched rank‐biserial (non‐parametric). Missingness ≤5% was handled via pairwise deletion; if >5% within an outcome family, multiple imputation by chained equations (predictive mean matching) was used and pooled estimates reported.

All enrolled participants completed both baseline and post‐intervention outcome assessments; accordingly, the primary endpoint analyses were performed on the full analytic cohort of 500 participants. Per‐protocol summaries were restricted to participants meeting the prespecified adherence threshold and were reported as secondary analyses. Missing assay values generated by marker‐specific quality‐control failure were handled at the marker level only; there was no post‐intervention outcome attrition requiring imputation of missing follow‐up visits. For variable‐level missingness below 5% within an outcome family, pairwise deletion was applied. If variable‐level missingness exceeded 5% within an outcome family, multiple imputation using predictive mean matching was prespecified. Sensitivity analyses included per‐protocol versus full‐cohort comparisons and re‐analysis excluding outliers (±3 SD from the group mean). No interim analyses were performed

## 3 Results

The enrolled cohort represented a healthy midlife sample with balanced sex and age‐band distribution, and baseline anthropometric, cardiovascular, and glucose values were consistent with the prespecified eligibility range. This profile supports interpretation of subsequent changes in a low‐risk, previously inactive cohort rather than a clinically impaired population (Table 1).

**TABLE 1:** Baseline demographics and health by sex and age group.

Variable	Total (*n* = 500)	Male (*n* = 268)	Female (*n* = 232)	Age 50–57 (*n* = 248)	Age 58–65 (*n* = 252)
Age (years)	57.1 ± 4.3	57.0 ± 4.4	57.2 ± 4.2	53.8 ± 1.9	60.2 ± 1.8
Body mass index (kg/m^2^)	24.6 ± 2.1	24.7 ± 2.0	24.5 ± 2.3	24.7 ± 2.0	24.5 ± 2.2
Resting heart rate (beats/min)	71.3 ± 8.2	70.9 ± 8.0	71.8 ± 8.4	70.7 ± 8.1	71.9 ± 8.3
Systolic blood pressure (mmHg)	126.4 ± 9.8	127.1 ± 9.9	125.6 ± 9.7	125.8 ± 9.5	127.0 ± 10.1
Diastolic blood pressure (mmHg)	78.2 ± 6.4	78.5 ± 6.3	77.9 ± 6.5	78.4 ± 6.2	78.0 ± 6.6
Fasting glucose (mmol/L)	5.42 ± 0.73	5.43 ± 0.70	5.41 ± 0.76	5.40 ± 0.68	5.44 ± 0.78

**FIGURE 3:**
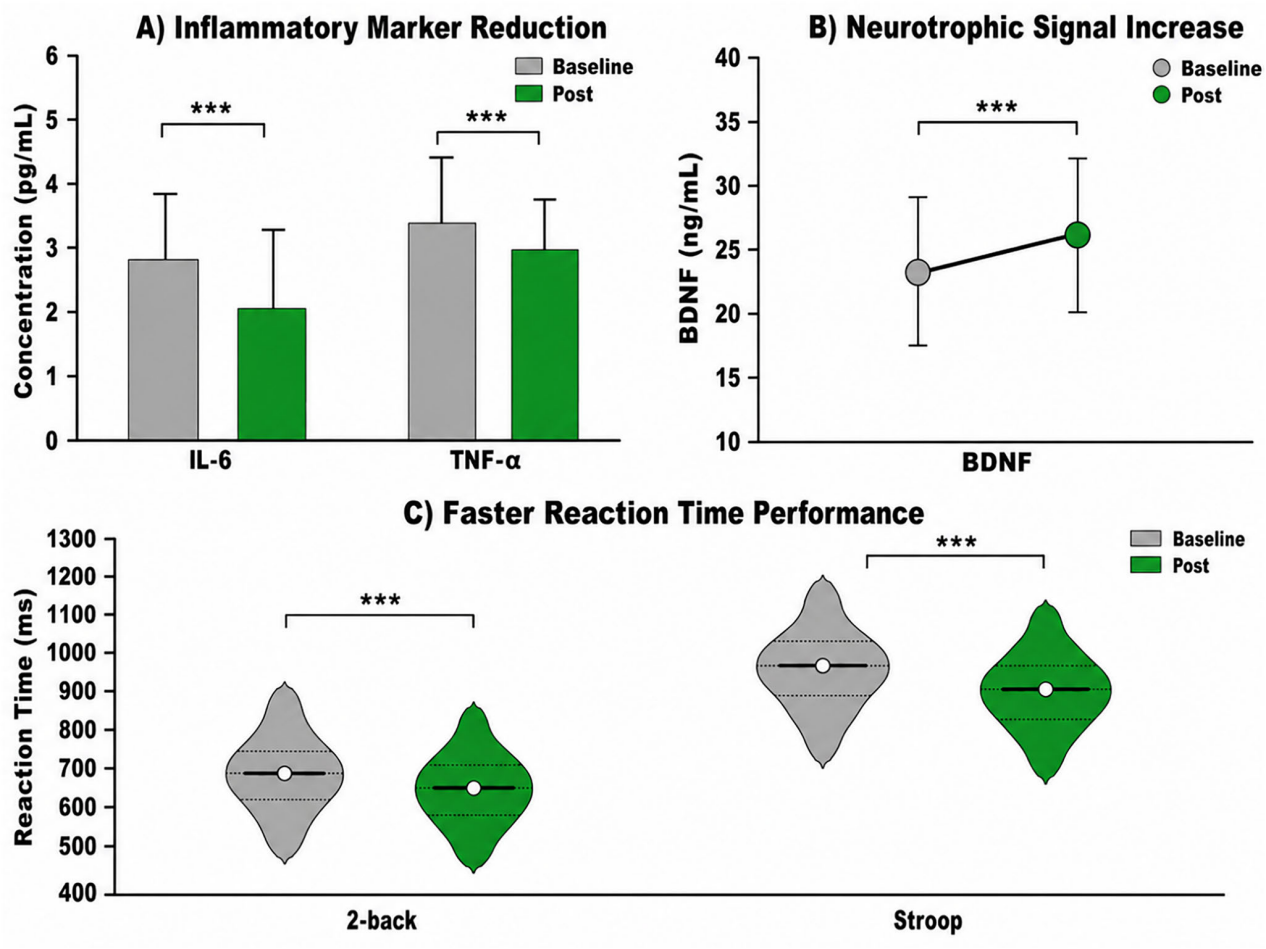
(A) Baseline and post‐intervention plasma IL‐6 and TNF‐α concentrations, shown as group means with SD error bars and paired‐comparison markers. (B) Baseline and post‐intervention plasma BDNF, shown separately because it is measured on a different scale from IL‐6 and TNF‐α. (C) Baseline and post‐intervention reaction‐time distributions for the 2‐back and Stroop tasks, with central tendency and spread shown for each condition. Data are mean ± SD for biomarker panels and distribution summaries for reaction‐time outcomes; *p < 0.05, **p < 0.01, *p < 0.001.

The baseline profile indicated a cognitively intact midlife cohort rather than a sample with marked executive impairment (Tables 2 and 3). After the 4‐week resistance‐training exposure, the biomarker profile shifted toward lower circulating inflammatory tone and higher peripheral neurotrophic signaling, with interleukin‐6 and tumor necrosis factor‐alpha decreasing while BDNF increased (Figure 3). Working‐memory and inhibitory‐control performance also shifted toward better task efficiency, reflected by improved two‐back accuracy, faster two‐back responses, higher Stroop accuracy, and reduced Stroop interference. The detailed pre–post estimates support a synchronized peripheral biomarker and executive‐task pattern within the full analytic cohort, while the magnitude of change remained modest and pre–post in nature.

**TABLE 2:** Pre–Post changes in circulating biomarker concentrations evaluated using paired‐sample tests.

Outcome	Pre (mean ± SD)	Post (mean ± SD)	Δ (Post–pre)	95% CI for Δ	*p* value
IL‐6 (pg/mL)	2.83 ± 1.60	2.09 ± 1.34	−0.74	−0.81 to −0.67	0.001
TNF‐α (pg/mL)	3.61 ± 1.07	3.12 ± 0.96	−0.49	−0.56 to −0.42	0.001
BDNF (ng/mL)	23.4 ± 5.6	26.1 ± 6.2	+2.7	2.3 to 3.1	0.001

**TABLE 3:** Changes in cognitive outcomes from baseline to post‐intervention evaluated using a paired‐sample *t*‐test.

Outcome	Pre	Post	Δ (Post–pre)	95% CI for Δ	*p* value
2‐Back accuracy (%)	83.7 ± 8.9	87.6 ± 7.2	+3.9	3.3 to 4.5	0.001
2‐Back RT (ms)	690 ± 89	652 ± 82	−38	−44 to −32	0.001
Omission errors (%)	5.4 ± 3.2	3.1 ± 2.5	−2.3	−2.6 to −2.0	0.001
Commission errors (%)	3.6 ± 2.7	2.2 ± 1.8	−1.4	−1.6 to −1.2	0.001
Stroop accuracy (%)	89.8 ± 7.4	91.9 ± 6.2	+2.1	1.5 to 2.7	0.001
Stroop RT congruent (ms)	734 ± 86	705 ± 79	−29	−33 to −25	0.001
Stroop RT incongruent (ms)	972 ± 89	916 ± 85	−56	−61 to −51	0.001
Stroop interference (ms)	238 ± 47	211 ± 41	−27	−31 to −23	0.001

Cognitive outcomes shifted toward more efficient working‐memory and inhibitory‐control performance after the 4‐week exposure period (Table 3). The two‐back pattern combined higher accuracy with faster correct responses, indicating improved task updating rather than a speed–accuracy trade‐off. Stroop outcomes showed the same direction of improvement, with higher accuracy and reduced interference, suggesting better inhibitory‐control efficiency. Declines in omission and commission errors further indicate fewer attentional lapses and fewer impulsive responses, although the changes remained modest within a cohort that already performed well at baseline.

Associations between biomarker change scores and cognitive change scores are presented in Table 4 as secondary analyses describing co‐occurrence across the intervention period. Change in BDNF showed the strongest associations, including positive associations with 2‐back accuracy and Stroop accuracy and an inverse association with 2‐back reaction time. IL‐6 change showed smaller associations with change in 2‐back accuracy and Stroop interference, whereas TNF‐α change showed weaker and less consistent associations across cognitive endpoints. These correlation analyses describe aligned within‐participant change but do not test mediation or mechanism.

**TABLE 4:** Correlations between change scores in biomarkers and cognition.

Predictor (Δ)	Δ 2‐Back accuracy (%)	Δ 2‐Back RT (ms)	Δ Stroop interference (ms)	Δ Stroop accuracy (%)
IL‐6 (pg/mL)	*r* = 0.18 (0.11–0.25), *p* = 0.003	*r* = −0.06 (−0.14–0.02), *p* = 0.124	*r* = 0.12 (0.04–0.20), *p* = 0.018	*r* = 0.03 (−0.05–0.11), *p* = 0.482
TNF‐α (pg/mL)	*r* = 0.09 (0.01–0.17), *p* = 0.037	*r* = −0.02 (−0.10–0.06), *p* = 0.678	*r* = 0.05 (−0.03–0.13), *p* = 0.246	*r* = 0.07 (−0.01–0.15), *p* = 0.091
BDNF (ng/mL)	*r* = 0.26 (0.18–0.33), *p* < 0.001	*r* = −0.17 (−0.25 to −0.09), *p* < 0.001	*r* = 0.21 (0.13–0.29), *p* < 0.001	*r* = 0.14 (0.06–0.22), *p* = 0.002

*Note: r* values are Pearson's *r* (or Spearman's ρ where non‐normal); values shown with 95% confidence intervals.

Abbreviations: Δ = post–pre; RT = reaction time.

**FIGURE 4:**
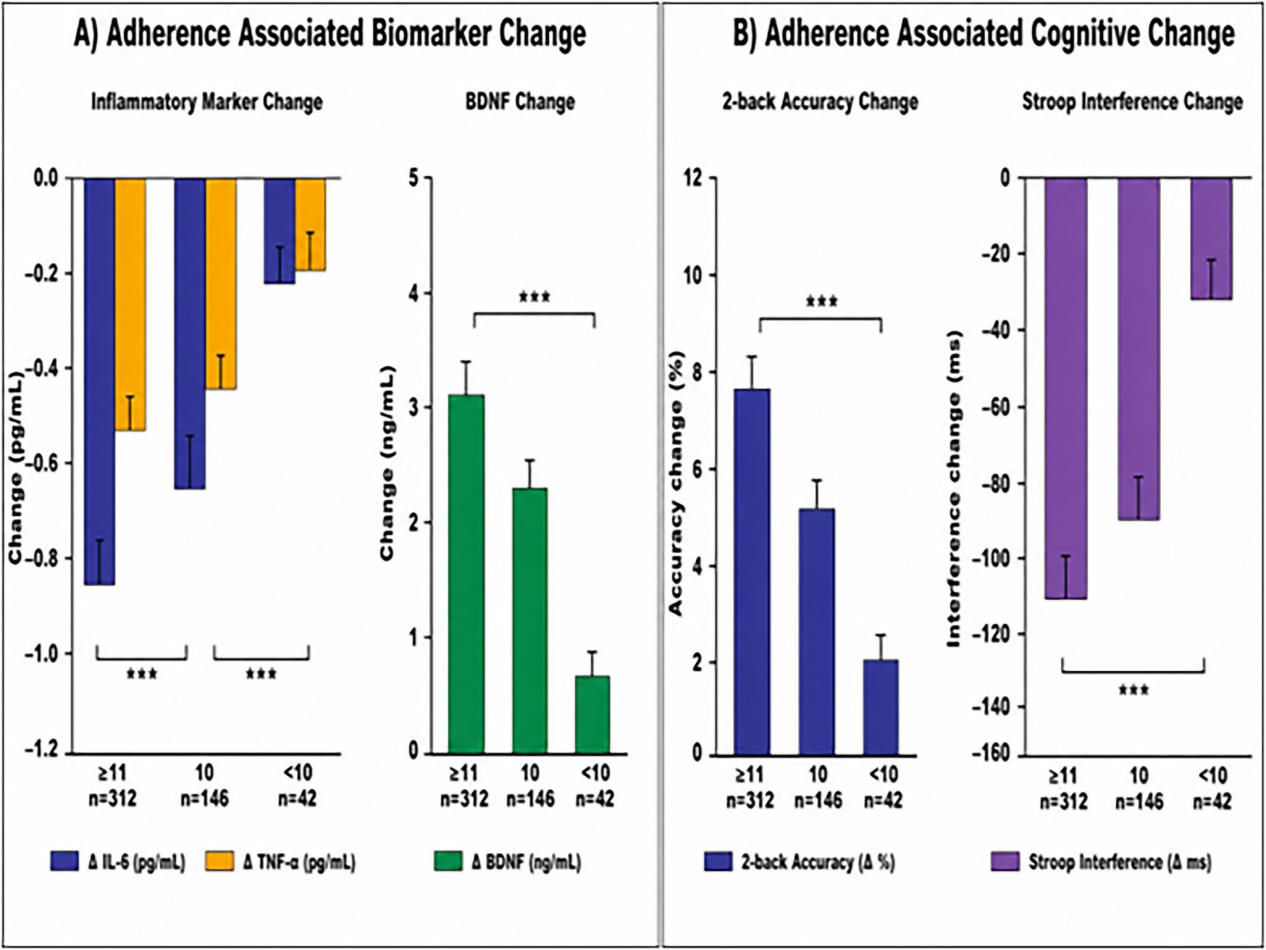
(A) Descriptive mean changes in plasma IL‐6, TNF‐α, and BDNF across adherence groups classified as ≥11 sessions, 10 sessions, and <10 sessions, showing larger biomarker shifts with higher session completion. (B) Descriptive mean changes in 2‐back accuracy and Stroop interference across the same adherence groups, showing stronger cognitive‐task improvement among participants with higher adherence. Data are mean ± SD; *p < 0.05, **p < 0.01, *p < 0.001.

Adherence‐stratified patterns indicated an exposure‐completion gradient, with the largest biomarker and executive‐task shifts among participants completing ≥11 sessions, intermediate shifts among those completing 10 sessions, and attenuated shifts among those completing <10 sessions (Table 5; Figure 4). The pattern was aligned across lower interleukin‐6 and tumor necrosis factor‐alpha, higher BDNF, improved two‐back accuracy, and reduced Stroop interference. Because adherence strata reflected observed session completion rather than randomized dose allocation, these findings describe secondary exposure‐completion differences rather than formal dose–response effects.

**TABLE 5:** Adherence‐stratified pre–post changes in biomarker and executive‐task outcomes.

Outcome Δ (post–pre)	≥11 Sessions (*n* = 312)	10 Sessions (*n* = 146)	<10 Sessions (*n* = 42)
Interleukin‐6 (pg/mL)	−0.85 ± 0.41	−0.66 ± 0.38	−0.21 ± 0.34
Tumor necrosis factor‐alpha (pg/mL)	−0.53 ± 0.28	−0.45 ± 0.25	−0.18 ± 0.22
Brain‐derived neurotrophic factor (ng/mL)	+3.1 ± 1.2	+2.3 ± 1.0	+0.7 ± 0.9
Two‐back accuracy (%)	+7.6 ± 3.4	+5.2 ± 2.7	+2.1 ± 2.3
Stroop interference (ms)	−110.4 ± 26.7	−88.2 ± 23.9	−31.6 ± 19.5

*Note*: Values are mean ± standard deviation. Adherence strata represent observed session completion and were not randomized dosage groups.

Linear regression using adherence count coded from 0 to 12 sessions showed that greater session completion was associated with larger biomarker change scores (Table 6). For IL‐6, the unstandardized coefficient was −0.067 with standardized β = −0.312 (95% CI −0.080 to −0.053; *p* = 0.0001). For TNF‐α, the unstandardized coefficient was −0.043 with β = −0.271 (95% CI −0.055 to −0.032; *p* = 0.0003). For BDNF, the unstandardized coefficient was +0.280 with β = +0.296 (95% CI +0.210 to +0.360; *p* = 0.0002). These regression results describe the association between session completion and biomarker change and are interpreted as secondary analyses within non‐randomized adherence variation.

**TABLE 6:** Linear regression of adherence count (0–12 sessions) on biomarker change scores.

Outcome (Δ)	B (Unstandardized)	β (Standardized)	95% CI	*t*	*p* value
Δ IL‐6 (pg/mL)	−0.067	−0.312	−0.080 to −0.053	−9.23	0.0001
Δ TNF‐α (pg/mL)	−0.043	−0.271	−0.055 to −0.032	−7.14	0.0003
Δ BDNF (ng/mL)	+0.280	+0.296	+0.210 to +0.360	+8.45	0.0002

Abbreviations: β = standardized coefficient; Δ = post–pre change; B = unstandardized regression coefficient; CI = confidence interval.

**FIGURE 5:**
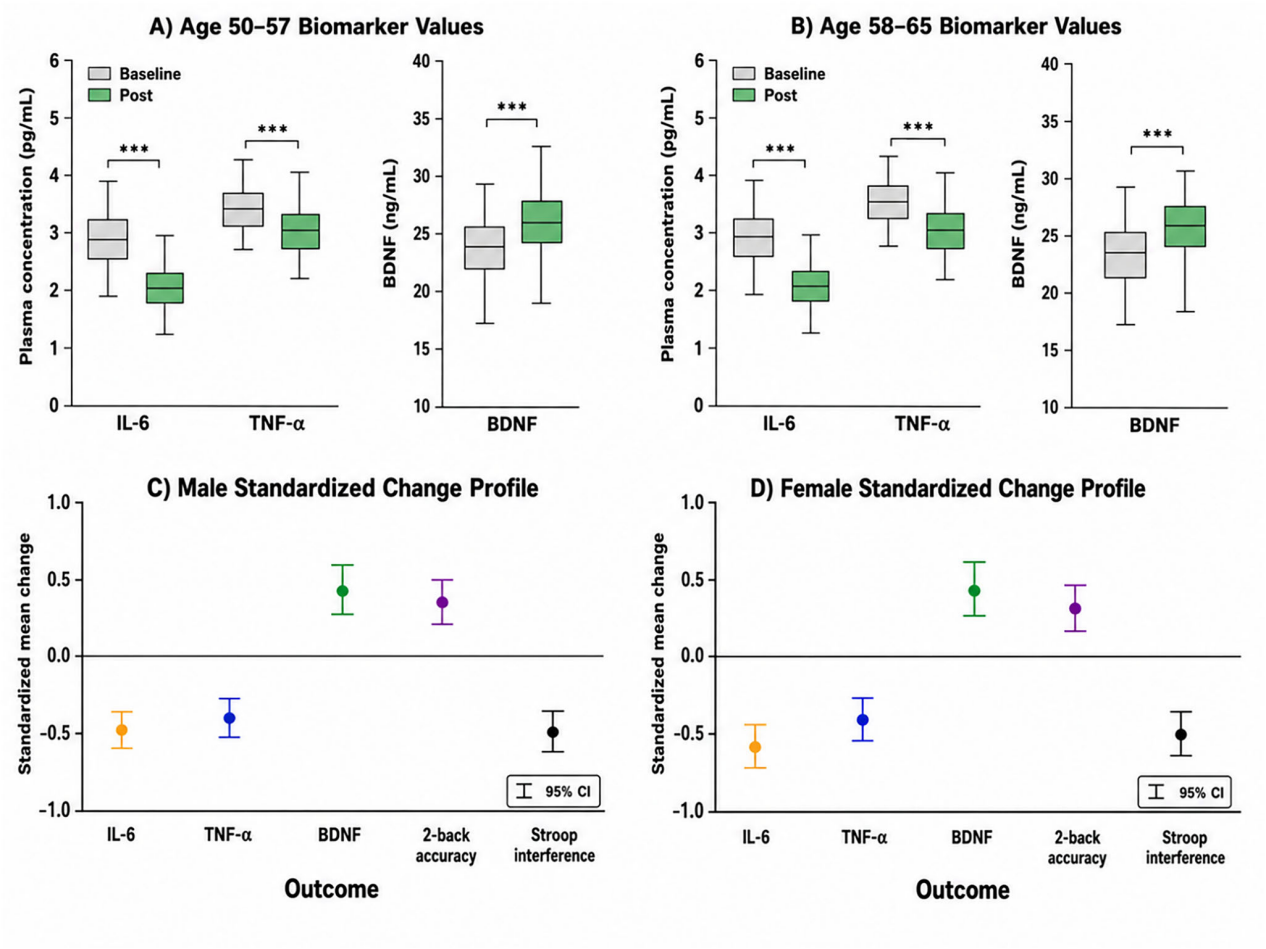
(A) Baseline and post‐intervention plasma IL‐6, TNF‐α, and BDNF values for participants aged 50–57 years, shown as biomarker distributions with paired‐comparison markers. (B) Baseline and post‐intervention plasma IL‐6, TNF‐α, and BDNF values for participants aged 58–65 years, presented using the same scale and format as in A. (C) Male subgroup standardized mean pre–post change across IL‐6, TNF‐α, BDNF, 2‐back accuracy, and Stroop interference, with error bars indicating 95% CI. (D) Female subgroup standardized mean pre–post change across the same biomarker and cognitive outcomes, with error bars indicating 95% CI. Data are shown as distributions for age‐stratified biomarker panels and standardized mean change ± 95% CI for sex‐stratified panels; *p < 0.05, **p < 0.01, * ***p* < 0.001**.

Age‐stratified biomarker changes followed the same direction in both age groups, with lower interleukin‐6 and tumor necrosis factor‐alpha and higher BDNF after the intervention (Figure 5A,B). Cognitive changes were also directionally aligned across age bands, indicating no clear age‐related separation in working‐memory or inhibitory‐control response (Table S3). Sex‐stratified patterns showed comparable standardized biomarker and cognitive shifts in male and female participants, supporting interpretation of subgroup findings as secondary descriptive patterns without evidence of differential response (Figure 5C,D).

**FIGURE 6:**
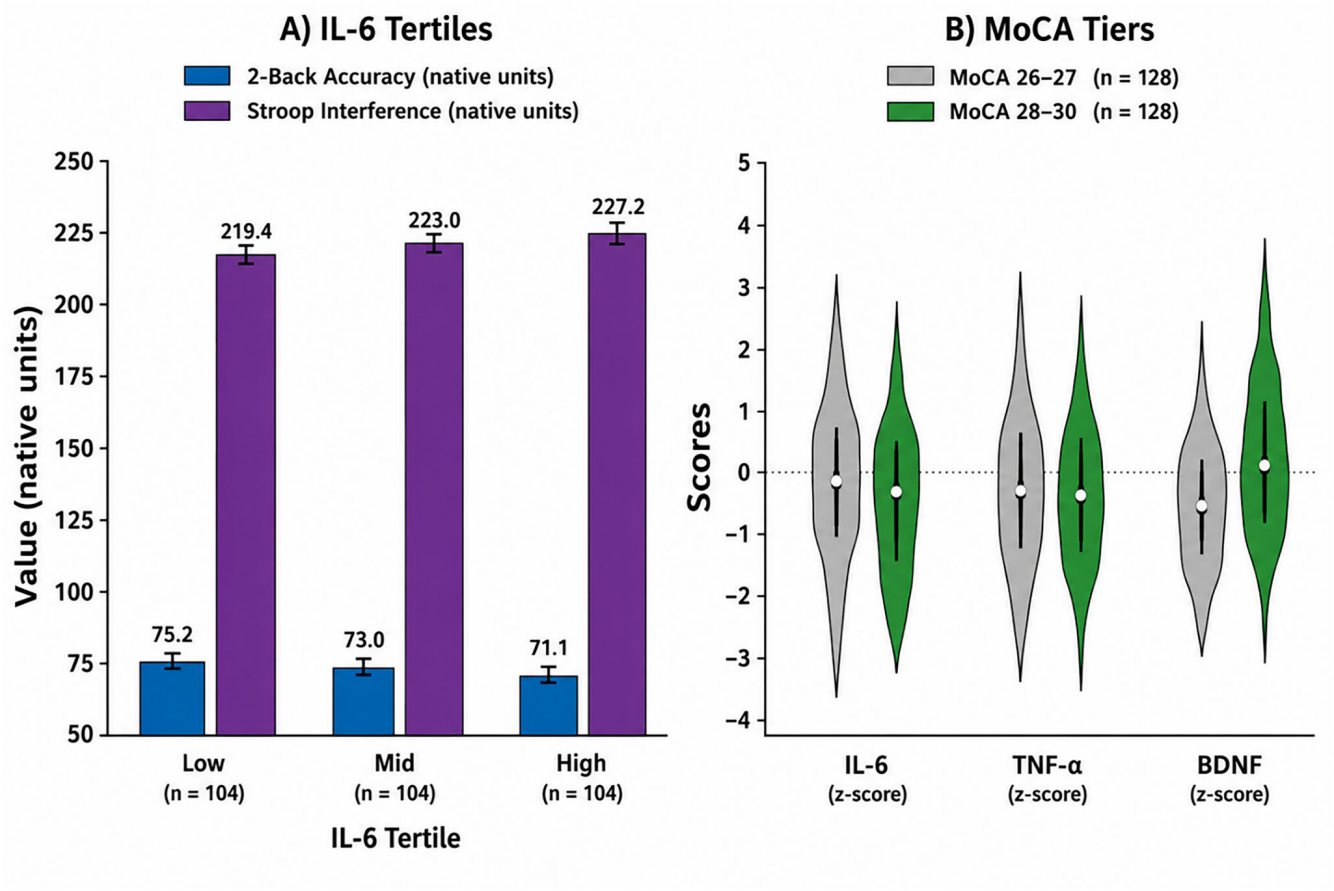
(A) Descriptive comparison of 2‐back accuracy and Stroop interference values across baseline IL‐6 tertiles classified as low, middle, and high, showing broadly similar cognitive profiles across inflammatory strata. (B) Distribution of standardized IL‐6, TNF‐α, and BDNF values across MoCA tiers 26–27 and 28–30, shown as violin plots with central tendency markers. Data are mean ± SD for tertile‐based cognitive values and distribution summaries for MoCA‐tier biomarker scores.

Pre–post cognitive change was similar across baseline IL‐6 tertiles, and between‐tertile comparisons were not statistically significant (Figure 6A,B). For 2‐back accuracy, the low tertile improved from 75.3% to 81.2% (+5.9), the middle tertile from 73.4% to 79.5% (+6.1), and the high tertile from 71.7% to 78.6% (+6.9), with no significant between‐group difference (*p* = 0.295). Error rates declined by approximately 3.0 points across tertiles (*p* = 0.374), Stroop accuracy improved by 1.9% to 2.7% (*p* = 0.198), and Stroop interference decreased by 17 to 20 ms (*p* = 0.132). Biomarker shifts were also similar across baseline MoCA tiers (Table S1), with IL‐6 decreasing by 0.74 pg/mL in both the 26–27 and 28–30 groups, TNF‐α decreasing by 0.48 and 0.49 pg/mL, and BDNF increasing by 2.8 and 2.6 ng/mL. Sensitivity analyses showed that the per‐protocol subset and full cohort yielded only small absolute differences in effect estimates, including IL‐6 change of −0.74 versus −0.68 pg/mL (*p* = 0.024), 2‐back accuracy change of +6.2% versus +5.7% (*p* = 0.046), and Stroop interference change of −19 versus −17 ms (*p* = 0.034), whereas other comparisons were not significant. Outlier‐excluded analyses remained consistent, with IL‐6 change of −0.70 pg/mL (95% CI −0.76 to −0.63), BDNF change of +2.6 ng/mL (95% CI 2.2 to 3.0), 2‐back improvement of +6.1%, and Stroop interference change of −18 ms. Missing‐data analyses also yielded near‐identical estimates, including IL‐6 change of −0.68 ± 0.49 versus −0.69 ± 0.48 (*p* = 0.782) and BDNF change of +2.5 ± 1.9 versus +2.4 ± 2.0 (*p* = 0.628), with cognitive estimates remaining closely aligned (Table S2).

## 4 Discussion

The main finding was that a 4‐week supervised RT exposure was associated with a coordinated within‐cohort pattern of lower plasma IL‐6 and TNF‐α, higher plasma BDNF, and modest improvement in working‐memory and inhibitory‐control task performance. The biological changes indicate a short‐interval shift in circulating inflammatory and neurotrophic markers, while the cognitive changes indicate improved task efficiency on the 2‐back and Stroop paradigms. The pattern was directionally consistent across age and sex strata and numerically stronger among participants with higher session completion. These findings are best interpreted as synchronized systemic biomarker and executive‐task changes observed under a standardized short‐cycle RT program. The single‐arm design does not establish comparative efficacy, intervention‐specific causality, mediation, or central neuroimmune mechanism. STRICC therefore functions as an organizing framework for peripheral and behavioral endpoints, not as an empirically verified pathway model.

IL‐6 and TNF‐α decreased after the 4‐week RT exposure, indicating a downward shift in resting plasma inflammatory markers across the intervention window. The 72‐h post‐session sampling interval supports interpretation as subacute resting concentrations, not acute post‐exercise cytokine excursions. This timing is important because high‐volume sessions can produce transient IL‐6 and TNF‐α elevations that resolve during recovery, whereas repeated moderate training may be associated with lower resting inflammatory tone (Enes et al. 2025; Wang et al. 2025). However, baseline IL‐6 and TNF‐α were already consistent with a healthy midlife cohort without marked inflammatory elevation. The observed reductions therefore indicate a modest downward shift within a low‐risk range, not normalization of abnormal inflammation or confirmed clinical anti‐inflammatory benefit. The larger changes among participants completing more sessions suggest exposure‐completion alignment, but this pattern remains observational because attendance may reflect motivation, scheduling flexibility, recovery capacity, or other participant‐level factors. Because the study lacked a comparator group, did not manipulate training volume, and measured biomarkers only at baseline and post‐intervention, the findings support short‐term systemic biomarker responsiveness under supervised RT exposure without establishing durable inflammatory remodeling, a minimum effective volume threshold, or training‐specific causality.

Plasma BDNF increased after the 4‐week RT exposure, with larger gains among participants completing more sessions. This pattern is consistent with peripheral neurotrophic responsiveness under repeated RT exposure, but it does not establish altered brain BDNF activity, synaptic plasticity, or central neurobiological adaptation. Plasma BDNF is sensitive to platelet handling, blood compartment effects, assay conditions, and peripheral release dynamics, so the finding should be interpreted as a circulating biomarker response aligned with executive‐task improvement. Prior studies have linked RT or exercise exposure with increased circulating BDNF and executive‐task gains, although protocols differ in modality, intensity, and population characteristics (Wang et al. 2025; Lee et al. 2025). Evidence from clinical or psychiatric samples also supports an association between lower BDNF and poorer cognitive status, but those findings are not direct comparators for healthy midlife adults (Gao 2025). In the present cohort, the adherence‐linked BDNF pattern provides a testable hypothesis that greater exposure completion may track with stronger peripheral neurotrophic signaling, requiring controlled studies with CNS‐aligned indices for mechanistic confirmation.

Cognitive performance shifted toward better task efficiency, with lower Stroop interference and higher 2‐back accuracy after the 4‐week RT exposure. These changes are consistent with improved inhibitory‐control and working‐memory task performance, but they remain behavioral outcomes from repeated Stroop and 2‐back testing. The single‐arm design cannot separate training‐associated change from practice effects, arousal regulation, expectancy, or repeated task exposure, especially in a cohort with high baseline cognitive status. Prior RT and multimodal exercise studies provide useful context for executive‐task change, although comparability is limited when protocols include cognitive loading, technology‐supported training, aerobic components, clinical samples, or different task batteries (Gonzalez‐Moreno et al. 2025; Mateluna‐Núñez et al. 2025; Mazreno and Taghian 2024). The present findings therefore support an association between short‐cycle RT exposure and improved executive‐task performance, without localizing the effect to prefrontal circuitry or specifying neurometabolic, vascular, or electrophysiological mechanisms.

Participants showed similar cognitive‐change patterns across baseline IL‐6 tertiles, with no clear separation between low, middle, and high inflammatory strata. This pattern suggests that baseline IL‐6 variation within this healthy cohort did not define distinct short‐term cognitive‐response profiles. Prior work supports a broader cytokine–cognition link, with higher IL‐6 associated with slower Stroop responses and lower executive scores (Dwojaczny et al. 2024), and higher pro‐inflammatory cytokines associated with weaker cognitive‐network function and lower 2‐back accuracy (Schmitz et al. 2025). Exercise‐based evidence also suggests that inflammatory modulation may coincide with memory improvement, although intervention components and populations differ from the present RT‐only cohort (Bonilla et al. 2024). In the present study, the IL‐6 tertile findings therefore indicate descriptive co‐variation across a low‐risk sample, not effect modification by baseline inflammation or mediation between cytokine change and task performance.

### 4.1 Practical Implications

These findings support the feasibility of a supervised short‐cycle RT program associated with measurable plasma biomarker and executive‐task changes in healthy midlife adults. The practical relevance lies in the structured delivery of a 4‐week protocol with high assessment completion, standardized supervision, and measurable adherence variation. The present design does not determine clinical prevention, long‐term durability, or superiority over aerobic, cognitive, or combined interventions. Implementation in wellness or occupational health settings should therefore be framed as a feasible short‐term monitoring model for RT exposure, plasma biomarker assessment, and task‐level cognitive follow‐up. Adherence thresholds may help describe exposure completion, but they should not be treated as confirmed dosage targets until tested in randomized or dose‐comparison designs.

### 4.2 Limitations and Future Research

This study was limited by the single‐arm pre–post design, absence of long‐term follow‐up, and restriction to healthy, previously inactive adults aged 50–65 years with low comorbidity burden and body mass index below 30 kg/m^2^. This cohort definition improved internal consistency for short‐interval biomarker interpretation but limits generalization to older adults, individuals with obesity, and clinical populations with higher inflammatory burden or different training tolerance. The absence of a randomized or concurrent control group also prevents separation of RT‐associated change from temporal effects, repeated testing, expectancy, or adherence‐related motivation.

Biomarker interpretation is also limited by the use of peripheral plasma measures. Plasma IL‐6 and TNF‐α do not quantify CNS inflammatory activity, and plasma BDNF is not a validated proxy for central synaptic plasticity. The study did not assess neuroimaging, cerebrospinal‐fluid biomarkers, blood–brain barrier permeability, or formal mediation pathways. Because baseline IL‐6 and TNF‐α were already within a healthy range, the observed reductions should be interpreted as downward shifts in circulating cytokines, not as clinically meaningful inflammatory normalization.

Future studies should use randomized comparator arms, repeated biomarker sampling during the intervention, and 3‐ and 6‐month follow‐up to determine durability and isolate training‐specific effects. CNS‐aligned measures and mediation models are also needed to test whether peripheral biomarker change, cognitive‐task improvement, and neuroimmune mechanisms are temporally or biologically linked.

## 5 Conclusion

Short‐term RT was associated with lower plasma IL‐6 and TNF‐α, higher plasma BDNF, and modest improvements in working memory and inhibitory control in cognitively healthy midlife adults. These findings reflect synchronized change over a 4‐week single‐arm exposure period and do not establish comparative efficacy, durable preventive benefit, or verified neuroimmune mechanism. The results indicate that a supervised RT‐only protocol can be studied as a feasible short‐cycle exposure linked to peripheral biomarker and executive‐task change. Consistent patterns across baseline cognitive and inflammatory strata, sensitivity analyses, and adherence categories support the stability of the observed associations, but the adherence findings remain descriptive because session completion was not randomized. Controlled trials with comparator arms, repeated biomarker sampling, central nervous system–aligned measures, and mediation frameworks are needed to test durability, mechanism, and clinical relevance.

## Author Contributions

**Yu Ziyan**: conceptualization, methodology, investigation, data curation, formal analysis, visualization, writing – original draft. **Chen Deqin**: methodology, investigation, resources, supervision, project administration, writing – review and editing. **Chen Fanyue**: conceptualization, methodology, validation, formal analysis, supervision, writing – review and editing.

## Funding

The authors have nothing to report.

## Conflicts of Interest

The authors declare no conflicts of interest.

## Supporting information




**Supporting Information**: brb371723‐sup‐0001‐SuppMat.docx

## Data Availability

All datasets generated and analyzed during the current study are available from the corresponding author upon reasonable request.
