# Supplementary material for: An Exploratory Study of the Short‐Term Association of Resistance Training With Circulating Inflammatory Biomarkers, Plasma Brain‐Derived Neurotrophic Factor, and Cognitive Function in Healthy Midlife Adults
Source: Brain Behav. 2026 Aug 25;16(8):e71723. doi: 10.1002/brb3.71723 (PMC13507518; doi:10.1002/brb3.71723)
Supplement: Supplementary file 1 [file BRB3-16-e71723-s001.docx]

**Figure S1.** (A) Distribution of biomarker change scores (Δ) for IL-6, TNF-α, and BDNF across the ≥11-session, 10-session, and <10-session adherence groups, with the dashed horizontal line indicating no change. (B) Supervised resistance-training facility used for intervention delivery. (C) Representative views of the participant training protocol and equipment layout used to standardize exercise exposure.

**Table S1. Pre–Post Changes Across Analysis Sets (Per-Protocol, Full Cohort, Outlier-Excluded)**

| **Variable** | **Per-Protocol Δ (95% CI)** | **Cohen’s d (PP)** | **Full Cohort Δ (95% CI)** | **Cohen’s d (FC)** | **Δ Difference (PP−FC)** | **p-value (PP vs FC)** | **Outlier-Excluded Δ (95% CI)** | **Cohen’s d (OE)** |
| --- | --- | --- | --- | --- | --- | --- | --- | --- |
| IL-6 (pg/mL) | −0.74 (−0.81 to −0.67) | 0.79 | −0.68 (−0.75 to −0.61) | 0.72 | +0.06 | 0.024 | −0.70 (−0.76 to −0.63) | 0.75 |
| TNF-α (pg/mL) | −0.49 (−0.56 to −0.42) | 0.61 | −0.46 (−0.52 to −0.39) | 0.58 | +0.03 | 0.071 | — | — |
| BDNF (ng/mL) | +2.7 (2.3 to 3.1) | 0.48 | +2.5 (2.1 to 2.9) | 0.44 | −0.2 | 0.138 | +2.6 (2.2 to 3.0) | 0.50 |
| 2-Back Accuracy (%) | +6.2 (5.7 to 6.7) | 0.63 | +5.7 (5.2 to 6.2) | 0.57 | −0.5 | 0.046 | +6.1 (5.5 to 6.7) | 0.62 |
| 2-Back Errors (%) | −3.0 (−3.4 to −2.6) | 0.58 | −2.6 (−3.0 to −2.3) | 0.53 | +0.4 | 0.051 | −3.0 (−3.5 to −2.5) | 0.58 |
| Stroop Accuracy (%) | +2.6 (2.3 to 2.9) | 0.49 | +2.3 (2.0 to 2.6) | 0.44 | −0.3 | 0.041 | +2.5 (2.0 to 3.0) | 0.46 |
| Stroop Interference RT (ms) | −19 (−21 to −17) | 0.54 | −17 (−19 to −15) | 0.49 | +2 | 0.034 | −18 (−20 to −16) | 0.52 |

*Notes:* PP = per-protocol (n=458); FC = full cohort (n=500); OE = outlier-excluded (±3 SD; n=448). Δ = post − pre. RT = reaction time. pg/mL = picograms/mL; ng/mL = nanograms/mL. “—” = not assessed in OE set.

**Table S2.** Missing-Data Sensitivity of Pairwise Deletion vs Multiple Imputation

| **Variable** | **Pairwise Δ Mean ± SD** | **Imputed Δ Mean ± SD** | **Δ Difference** | **p-value** |
| --- | --- | --- | --- | --- |
| IL-6 (pg/mL) | −0.68 ± 0.49 | −0.69 ± 0.48 | +0.01 | 0.782 |
| BDNF (ng/mL) | +2.5 ± 1.9 | +2.4 ± 2.0 | −0.1 | 0.628 |
| 2-Back Accuracy (%) | +5.7 ± 3.4 | +5.6 ± 3.5 | −0.1 | 0.661 |
| 2-Back Errors (%) | −2.6 ± 2.8 | −2.7 ± 2.9 | +0.1 | 0.702 |
| Stroop Accuracy (%) | +2.3 ± 2.3 | +2.2 ± 2.4 | −0.1 | 0.639 |
| Stroop Interference RT (ms) | −17 ± 10 | −17 ± 11 | 0.0 | 0.990 |

*Notes:* Δ = post − pre; SD = standard deviation; RT = reaction time. Results indicate minimal sensitivity to missing-data handling.

**Table S3:** Age-stratified pre–post changes in biomarker and executive-task outcomes

| **Outcome Δ (Post–Pre)** | **Age 50–57 (n = 248)** | **Age 58–65 (n = 252)** | **Between-age comparison** |
| --- | --- | --- | --- |
| Interleukin-6 (pg/mL) | −0.74 ± 0.50 | −0.74 ± 0.52 | p = 0.941 |
| Tumor necrosis factor-alpha (pg/mL) | −0.46 ± 0.31 | −0.52 ± 0.34 | p = 0.118 |
| Brain-derived neurotrophic factor (ng/mL) | +2.7 ± 1.8 | +2.5 ± 1.9 | p = 0.286 |
| Two-back accuracy (%) | +4.0 ± 3.0 | +3.8 ± 3.1 | p = 0.462 |
| Two-back reaction time (ms) | −37 ± 31 | −39 ± 33 | p = 0.508 |
| Stroop accuracy (%) | +2.2 ± 2.8 | +2.0 ± 2.9 | p = 0.437 |
| Stroop interference (ms) | −28 ± 24 | −26 ± 25 | p = 0.391 |

Note: Values are mean ± standard deviation. Between-age comparisons summarize differences in change scores between the 50–57 and 58–65 year groups.
